# Brain Serotonin Signaling Does Not Determine Sexual Preference in Male Mice

**DOI:** 10.1371/journal.pone.0118603

**Published:** 2015-02-23

**Authors:** Mariana Angoa-Pérez, Nieves Herrera-Mundo, Michael J. Kane, Catherine E. Sykes, John H. Anneken, Dina M. Francescutti, Donald M. Kuhn

**Affiliations:** 1 Research & Development Service, John D. Dingell VA Medical Center, Detroit, Michigan, United States of America; 2 Department of Psychiatry & Behavioral Neurosciences, Wayne State University School of Medicine, Detroit, MI 48201, United States of America; Duke University, UNITED STATES

## Abstract

It was reported recently that male mice lacking brain serotonin (5-HT) lose their preference for females (Liu et al., 2011, Nature, 472, 95–100), suggesting a role for 5-HT signaling in sexual preference. Regulation of sex preference by 5-HT lies outside of the well established roles in this behavior established for the vomeronasal organ (VNO) and the main olfactory epithelium (MOE). Presently, mice with a null mutation in the gene for tryptophan hydroxylase 2 (TPH2), which are depleted of brain 5-HT, were tested for sexual preference. When presented with inanimate (urine scents from male or estrous female) or animate (male or female mouse in estrus) sexual stimuli, TPH2-/- males show a clear preference for female over male stimuli. When a TPH2-/- male is offered the simultaneous choice between an estrous female and a male mouse, no sexual preference is expressed. However, when confounding behaviors that are seen among 3 mice in the same cage are controlled, TPH2-/- mice, like their TPH2+/+ counterparts, express a clear preference for female mice. Female TPH2-/- mice are preferred by males over TPH2+/+ females but this does not lead to increased pregnancy success. In fact, if one or both partners in a mating pair are TPH2-/- in genotype, pregnancy success rates are significantly decreased. Finally, expression of the VNO-specific cation channel TRPC2 and of CNGA2 in the MOE of TPH2-/- mice is normal, consistent with behavioral findings that sexual preference of TPH2-/- males for females is intact. In conclusion, 5-HT signaling in brain does not determine sexual preference in male mice. The use of pharmacological agents that are non-selective for the 5-HT neuronal system and that have serious adverse effects may have contributed historically to the stance that 5-HT regulates sexual behavior, including sex partner preference.

## Introduction

Serotonin (5-HT) axons innervate virtually all areas of the nervous system from the B1–B9 clusters of neuronal cell bodies located in the mesencenphalon [2]. In its function as a neurotransmitter, 5-HT is thought to be extremely important in mediating a very large number of behavioral and physiological processes to include sleep [3,4], aggression [5,6], aging [7] and control of food intake and body weight [8]. In addition, dysfunction in the 5-HT neuronal system has been linked to numerous neuropsychiatric conditions including depression and suicide [9], anxiety [10] and obsessive-compulsive disorder [11]. A role for 5-HT in sexual function emerged from early studies showing that depletions of the neurotransmitter resulted in hypersexuality [12,13,14,15,16,17,18]. Recent experiments using mice with reductions in the number of 5-HT neurons or in 5-HT itself via null mutations in the genes for LIM homeobox transcription factor 1-b (*Lmx1b*) or tryptophan hydroxylase 2 (TPH2), respectively, suggested for the first time that 5-HT regulates sexual preference [1,19]. In these studies, male [1] and female [19] *Lmx1b-/-* and TPH2-/- mice lost their preference for mice of the opposite sex and this preference could be reinstated by pharmacological replenishment of 5-HT.

Interest in TPH2 was reinvigorated when it was discovered that this enzyme existed in two distinct forms [20]. TPH was historically considered to be a single gene product and the only form of this enzyme [21]. Now it is known that TPH1 and TPH2 are distinct gene products and TPH1 is the non-neuronal form while TPH2 is expressed selectively in neurons [22]. Because TPH2 is the initial and rate limiting enzyme in the biosynthesis of 5-HT, mice in which the TPH2 gene has been ablated lack brain 5-HT. A surprisingly large number of research groups have independently created TPH2-/- mice [23,24,25,26,27] and these animals display a number of very interesting physiological and behavioral phenotypes. For instance, TPH2-/- mice show growth retardation and altered autonomic control [27,28], modifications in bone mass accrual and appetite [25], impaired exercise-induced hippocampal neurogenesis [29] and reduced ventilatory response to intermittent hypoxia [30]. In view of the presumed essential role of 5-HT in the development of the CNS [31,32,33,34], it has come as something of a surprise that general brain development [35] and elaboration of the 5-HT neuronal system in TPH2-/- mice is relatively normal [23,28,36,37,38,39] and 5-HT neurons in these mice, while lacking 5-HT, retain their characteristic electrophysiological properties [37]. From a behavioral perspective, TPH2-/- mice show intense compulsivity and impulsivity [39], social communication deficits [40] and exaggerated aggression with decreased levels of anxiety-like behavior [39,41].

In the course of developing and maintaining our colony of TPH2-/- mice we have noted, apart from occasional bouts of aggression, relatively normal sexual approach and courtship behavior in mating pairs of TPH2-/- mice. The results of studies suggesting that TPH2-/- mice lose their sexual preference [1,19] prompted us to examine preference behavior in our TPH2-/- mice in more depth. We report presently that male TPH2-/- mice prefer female mice of either genotype (TPH2+/+ or TPH2-/-) over male mice. It also appears that female TPH2-/- mice are more sexually approachable than TPH2+/+ females but this does not lead to greater fertility. Last, markers of pheromone detection in the VNO (TRPC2) and odorant signal transduction in the MOE (CNGA2) of TPH2-/- males were not different from their wild-type counterparts.

## Materials and Methods

### Animals

TPH2−/− mice were generated by deleting exon 1 of the Tph2 gene as described [42] and were on a mixed C57BL/6-Sv129 background. Genome scanning analysis by the Jackson Laboratory (Bar Harbor, ME) revealed that our strain background was ~95% C57BL/6. Mice were housed in a temperature controlled colony room with a 12/12 h light/dark schedule and had access to food and water *ad libitum*. All mice were socialized (i.e., group housed), sexually naïve and between 8–14 weeks of age. All behavioral tests were observed and recorded by two independent observers. The Institutional Animal Care and Use Committee of Wayne State University approved the animal care and experimental procedures (Permit Number: A3310–01) and all procedures are in compliance with the NIH *Guide for the Care and Use of Laboratory Animals*.

### Urine Preference Test

Filter paper (15.5 × 26.5 cm) was cut to cover the entire bottom of a small mouse cage (16 × 27 × 13 cm). Urine was collected from TPH2+/+ males and TPH2+/+ and TPH2−/− female mice in estrus. Estrus was confirmed by microscopic examination of vaginal smears and is characterized by clusters of cornified squamous epithelial cells [43]. In order to minimize individual variability, urine from mice of the same genotype and sex was pooled and stored at -20°C until used. Briefly, 10 μl of urine was pipetted onto the filter paper squares placed at opposite ends of the test cage. Males of either genotype were presented with a choice between urine from a TPH2+/+ male versus urine from either a TPH2+/+ or TPH2-/- female. Immediately after the paper was impregnated with urine, a male TPH2+/+ or TPH2-/- mouse was introduced into the center of test cage. The amount of time spent sniffing each spot was recorded during a 5 min test period.

### Sexual Preference Behavior

**Sexual interactions between 2 mice**. This test was performed to determine male interactions with a single, receptive female. Male mice of either genotype were placed individually into a small test cage (16 × 27 × 13 cm) for 30 minutes and immediately thereafter, a TPH2+/+ or TPH2−/− female in estrus was placed into the test cage with the male. The latency to mount and the number of mounts, attacks and intromissions were counted in a 15-min test period.

**Sexual interactions among 3 mice**. This test aimed to determine whether male mice express a sexual preference when presented simultaneously with a choice between a single male and a single receptive female mouse or a choice between two receptive female mice. Male TPH2+/+ or TPH2-/- mice (i.e., the resident) were placed individually into a small test cage (16 × 27 ×13 cm) for 30 minutes and then two intruder mice were introduced into the cage simultaneously. In one format, the intruder mice were a male TPH2+/+ mouse and a female TPH2+/+ mouse in estrus. In a second format, the intruder mice were a receptive female TPH2+/+ mouse and a receptive female TPH2-/- mouse. The latency and number of mounts, attacks and intromissions were scored for a 15-min period. In order to differentiate between TPH2+/+ and TPH2−/− female intruder mice, tails were coded distinctly with a permanent marker. Because of the complexity of the interactions among 3 mice in the same cage simultaneously (i.e., intruder mice interacting with each other as well as with the resident mouse), we modified the test to restrict access of the resident mouse with the intruder mice by placing the intruders in wire cups as used in the three chamber social approach test in the study of autism-like behaviors in rodents [40,44,45]. These cups allow olfactory, auditory, visual and limited tactile contact with the intruder mice but prevent mounting, fighting, grooming and other direct forms of physical contact among the 3 mice being tested. Male residents were scored for the percentage of time spent sniffing and investigating the “caged” intruders in a 15 min test period.

### Mating success

Females were housed for 21 days with males in the following genotype combinations, with the female of the pairing listed first: TPH2+/+ x TPH2+/+; TPH2+/- x TPH2+/+, TPH2+/- or TPH2-/-, and TPH2-/- x TPH2+/- or TPH2-/-. Matings that would produce litters that were 100% heterozygous were not carried out. We had no experimental plan for the use of excess numbers of TPH2+/- mice, so these matings were not justifiable from the perspective of animal use guidelines. At the end of this two week period, males were removed and from this time forward, females remained singly housed until giving birth or until the passage of an additional 21 days (i.e., unsuccessful pregnancy). A mating was scored as fertile only when a litter was born.

### Determination of TRP2 and CNGA2 protein levels by immunoblotting

Male mice were sacrificed by decapitation and the VNO and MOE were dissected and stored frozen at −80°C until assayed. Frozen tissue was disrupted by sonication in 1% SDS at 95°C and insoluble material was sedimented by centrifugation. Protein was determined by the bicinchoninic acid method and equal amounts of protein (70 μg/lane) were resolved by SDS-polyacrylamide gel electrophoresis and then electroblotted to nitrocellulose. Blots were blocked in Odyssey Infrared Imaging System blocking buffer (LI-COR Biosciences, Lincoln, NE, USA) for 1 h at room temperature. Primary antibodies against TRPC2 (1:500, Novus Biologicals, Littleton, CO), CNGA2 (1:500, Abcam, Cambridge, MA) or GAPDH (1:2000, Sigma-Aldrich, St. Louis, MO) were added to blots and allowed to incubate for 16 h at 4°C. Blots were washed 3X in Tris-buffered saline with 0.1% Tween 20 and once with 1X PBS to remove unreacted antibodies and then incubated with fluorescent IRDye anti-IgG secondary antibodies (1:4000) in the dark for 1 h at room temperature. Immunoreactive bands were visualized by enhanced chemiluminescence and the relative densities of TRPC2-, CNGA2- and GAPDH-reactive bands were determined by imaging with an CLx Odyssey Image Station (LI-COR, Biosciences, Lincoln, NE) and quantified using ImageJ software (NIH). Expression levels of TRPC2 and CNGA2 were normalized to GAPDH levels.

### Statistical Analysis

Data from the urine preference test and sexual preference behavior when using 3 mice simultaneously were analyzed by two-way ANOVA and post hoc comparisons were carried out using Bonferroni’s multiple comparison test. Data from sexual preference behavior in one-on-one pairings were carried out using one-way ANOVA and post hoc comparisons were made using Tukey’s multiple comparison test. Tests of mating success among pairings of genotypes were analyzed using a two-sided Fisher’s Exact test. Student’s t-tests were performed to analyze the normalized levels of TRPC2 and CNGA2 expression. Values of p < 0.05 were deemed statistically significant. All statistical analyses were carried out using GraphPad Prism version 6.02 for Windows, GraphPad Software, San Diego, CA, www.graphpad.com.

## Results

### Male TPH2-/- mice prefer the scent of female urine/pheromones over that of males

**Fig 1:**
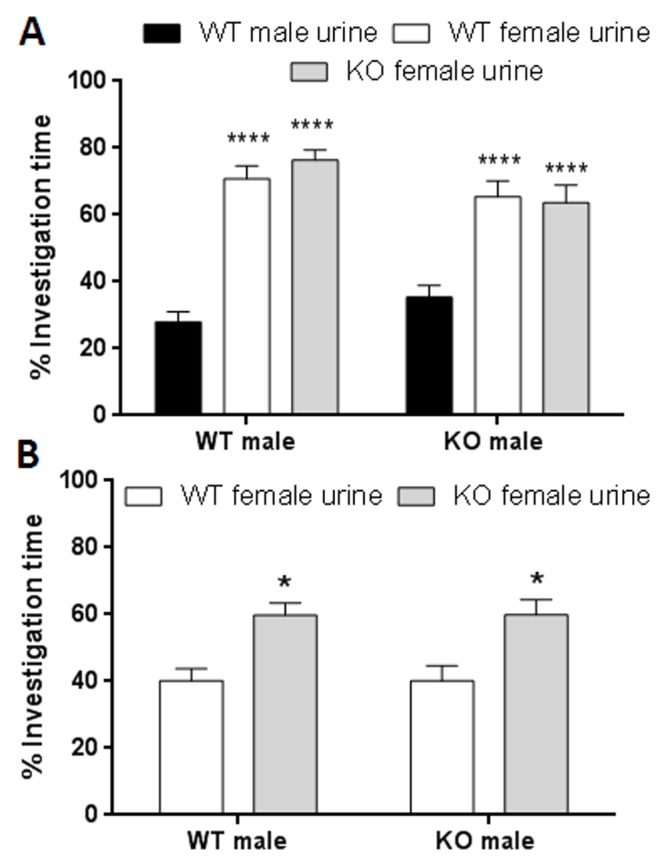
Male preference for urine scents from TPH2+/+ males versus TPH2+/+ females or TPH2-/- females. (A) % time spent investigating urine scents by TPH2+/+ (WT) or TPH2-/- (KO) males when given the choice between urine from a WT male versus urine from a receptive WT or KO female, (B) % time investigating urine scents by WT or KO males when given the choice between scents from a receptive WT female versus a receptive KO female. Data are expressed as the mean ± SEM for groups containing 9 WT and 10 KO males. Symbols indicate significant difference from the WT male. * p < 0.05; **** p < 0.0001.

TPH2+/+ and TPH2-/- male mice were exposed to urine samples from TPH2+/+ males versus urine from either a TPH2+/+ or a TPH2-/- female. The results in Fig. 1A indicate that males of both genotypes preferred the scent of female urine (either TPH2+/+ or TPH2-/-) over that of males (F_2,58_ = 53.63, p < 0.0001). An effect of male genotype was not present and the urine scent x genotype interaction was likewise not significant. Post hoc comparisons showed a significant preference by TPH2+/+ and TPH2-/- males for the urine scent of either female genotype over that of TPH2+/+ male urine (p < 0.0001 for each). When males of either genotype were given the choice of urine scents from female TPH2+/+ versus female TPH2-/-, a significant preference (F_1,34_ = 21.92, p < 0.0001) was seen such that both TPH2+/+ males and TPH2-/- males displayed a significant preference for the urine scent of TPH2-/- females over that of TPH2+/+ females (p < 0.05 for both). These data show that male TPH2-/- and TPH+/+ mice have the same preference for females when given a choice of male and female inanimate sexual stimuli. Males of both genotypes also show a significant preference for urine scents of TPH2-/- females over scents from TPH2+/+ females.

### Male TPH2-/- mice prefer female mice over male mice in one-on-one encounters

**Fig 2:**
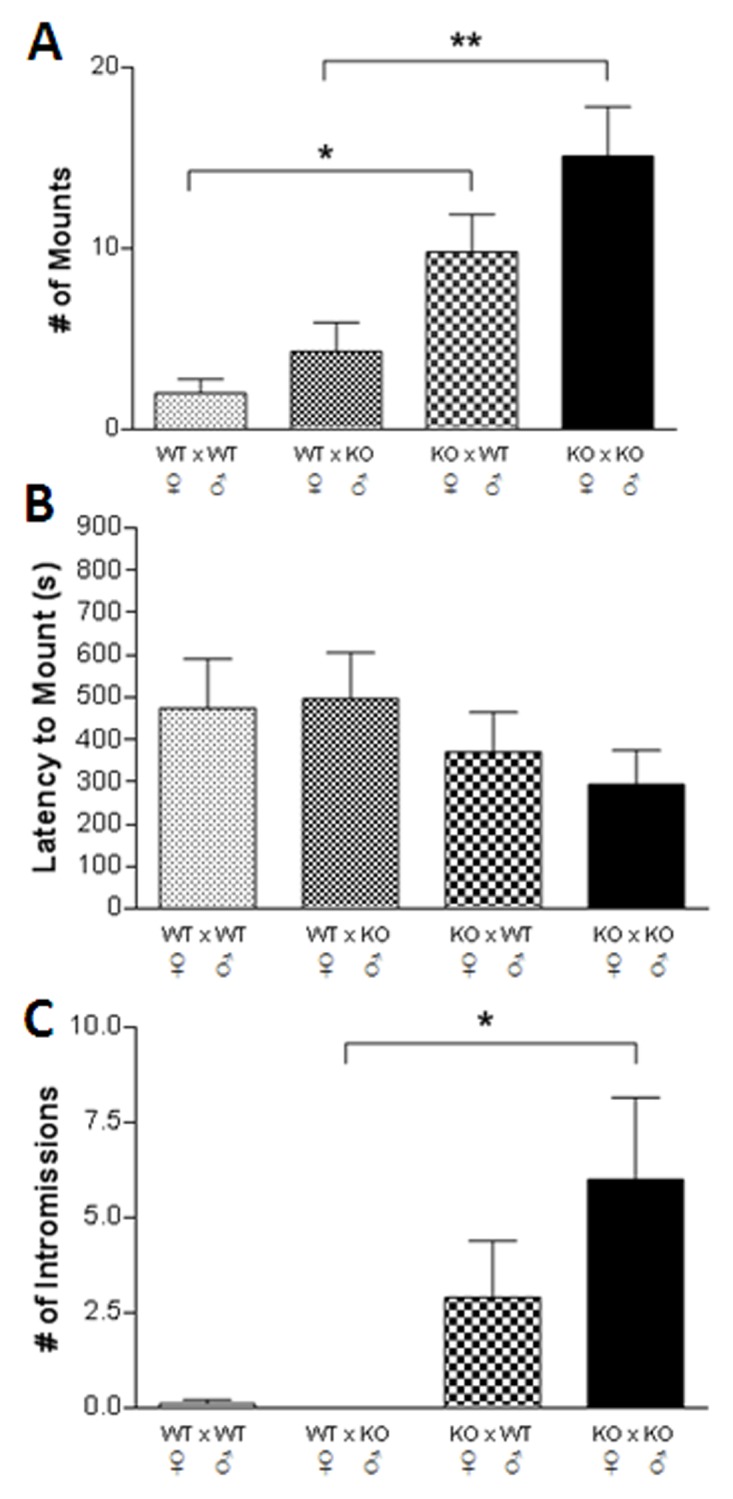
Male sexual preference in one-on-one encounters. (A) # of mounts when a single WT or KO male was exposed to a single, receptive WT or KO female, (B) latency to mount by WT and KO males when exposed to a receptive WT or KO female, (C) # of intromissions by WT and KO males when exposed to a receptive WT or KO female. Data are expressed as the mean ± SEM for groups containing 10 WT and 10 KO males. Symbols indicate significant difference from the indicated comparison. * p < 0.05; ** p < 0.01.

The results from pairings of individual male and female mice are presented in Fig. 2. It can be seen in Fig. 2A that a significant preference (F_3,35_ = 8.60, p < 0.0002) for mounting TPH2-/- females over TPH2+/+ females was shown by both TPH2+/+ (p < 0.05) and TPH2-/- (p < 0.01) males. TPH2+/+ and TPH2-/- males did not differ from each other in the number of mounts when paired with a female of either genotype. The latency to mount did not vary significantly in any of these pairings (Fig. 2B). The number of intromissions achieved by male mice is presented in Fig. 2C and it can be seen that the effect of pair genotype was significant (F_3,35_ = 4.47, p < 0.009). Male TPH2-/- mice had significantly more intromissions with TPH2-/- females by comparison to TPH2+/+ females (p < 0.05). While TPH2+/+ males showed a trend toward more intromissions with TPH2-/- females versus TPH2+/+ females, this difference did not reach significance. As seen for mounts, TPH2+/+ males did not differ from TPH2-/- males in the number of intromissions with females of either genotype. We carried out male-on-male pairings but found that sexually-directed behaviors such as mounting and intromissions were completely displaced by overt aggression on the part of TPH2-/- males toward their cage partner (i.e., either TPH2+/+ or TPH2-/- male), so it was not possible to determine male sexual preference in testes-intact TPH2-/- males. Future studies will carry out male-on-male pairings in castrated TPH2-/- mice in an attempt to reduce aggressive behavior [46,47].

### Male TPH2+/+ and TPH2-/- mice do not have a sex preference when exposed to a male and female mouse simultaneously

**Fig 3:**
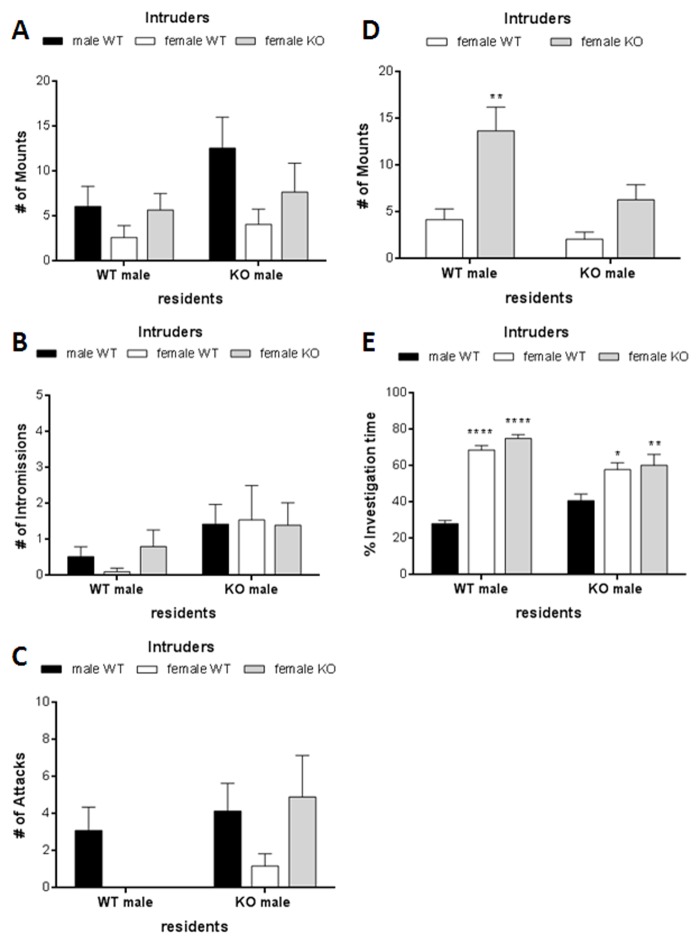
Male sexual preference when exposed to two mice simultaneously. (A) # of mounts by WT or KO males when exposed simultaneously to a WT male versus a WT female or to a WT male versus a KO female, (B) # of intromissions by WT and KO males when exposed simultaneously to two mice as described in A, (C) # of aggressive attacks by WT and KO males when exposed simultaneously to two mice as described in A, (D) # of mounts by WT or KO males when exposed simultaneously to a receptive WT female and a receptive KO female, (E) % investigation time when WT or KO males were exposed simultaneously to a “caged” WT male versus a “caged” WT female or to a “caged” WT male versus a “caged” KO female. Data are expressed as the mean ± SEM for groups containing 6–11 WT and 10 KO males. Symbols indicate significant difference from the WT control (female or male). * p < 0.05; ** p < 0.01; **** p < 0.0001.

We followed the approach of Liu and colleagues [1] to determine if male mice of either genotype would show a sex preference when presented with a male and a receptive female mouse simultaneously. Resident TPH2+/+ or TPH2-/- mice, when allowed to interact with a male TPH2+/+ mouse along with a female TPH2+/+ mouse in estrus showed the same number of mounts on the male and female intruders (Fig. 3A). Likewise, when the paradigm was changed to include an intruding TPH2-/- female and a TPH2+/+ male, the number of mounts by the resident TPH2+/+ or TPH2-/- males was the same (i.e., no preference for male or female), as shown in Fig. 3A. Neither TPH2+/+ nor TPH2-/- males showed a sex preference for the first mount of an intruder mouse (data not shown). We also scored the number of intromissions in these pairings and while low in number, a difference between TPH2+/+ and TPH2-/- males was not observed when the intruding cohort female was either TPH2+/+ or TPH2-/- (Fig. 3B). Fig. 3C shows that resident males of either genotype attacked both male and female TPH2+/+ intruders although attacks by TPH2+/+ males on females of either genotype did not occur. TPH2-/- males showed more attacks on all intruders (male and female) by comparison to TPH2+/+ mice, but this effect was not significant. Fig. 3D shows that the genotypes of both the intruding female (F_1,36_ = 17.30, p = 0.0002) and resident male (F_1,36_ = 8.31, p = 0.006) were significant for the number of mounts. The intruder genotype x resident genotype interaction was not significant. TPH2+/+ resident males mounted the TPH2-/- female significantly more than the TPH2+/+ female (p < 0.01), and while TPH2-/- males showed a trend to greater numbers of mounts on the TPH2-/- female versus the TPH2+/+ female, this effect did not reach statistical significance. Using the approach of Liu and colleagues [1] by allowing 3 mice to interact freely in the same cage simultaneously, it appears that both TPH2+/+ and TPH2-/- males lost their preference for females of either genotype.

Because of the complexity of the interactions among 3 mice in the same cage simultaneously, we modified the test to restrict access of the intruder mice to the resident mouse by placing the intruders in wire cups [40,44,45]. These cups allow limited contact with the intruder mice but prevent mounting, fighting, grooming and other direct forms of physical contact among the 3 mice being tested. Fig. 3E shows that the genotype of intruder mice significantly influenced the percentage of investigation time spent by resident males of both genotypes (F_2,40_ = 63.40, p < 0.0001). The interaction between intruder x resident genotype was also highly significant (F_2,40_ = 10.71, p = 0.0002). When placed into a cage containing male and female TPH2+/+ intruders within wire cups, male TPH2+/+ (p < 0.0001) and TPH2-/- mice (p < 0.05) showed a significant preference for investigating female TPH2+/+ mice over TPH2+/+ males. Fig. 3E also indicates that both male TPH+/+ (p < 0.0001) and TPH2-/- mice (p < 0.01) showed a significant preference for investigating female TPH2-/- mice over TPH2+/+ males. Neither TPH2+/+ nor TPH2-/- males showed a sex preference for the first approach to an intruder mouse (data not shown). Therefore, when the confounding behaviors associated with testing 3 mice simultaneously in a social contact setting (to include a female in estrus) are controlled, TPH2-/- males like their TPH2+/+ counterparts clearly prefer female mice of either genotype over a male.

### Mating success rates in TPH2-/- females

In light of the data above showing that TPH2-/- females in estrus seemed to be preferred over TPH2+/+ females by males of both genotypes (Figs. 1 and 2), we predicted that this preference could contribute to higher rates of mating success in TPH2-/- females. Therefore, we compared mating success rates of TPH2-/- females to TPH2+/+ and TPH2+/- females when paired with males of each genotype. For the sake of clarity, the statistical comparisons of mating outcomes among all rows and the levels of significance are presented in the legend of Table 1. The results in Table 1 show that pairings of TPH2+/+ females and males or TPH2+/- females and males resulted in the highest rates of pregnancy success (89% and 97%, respectfully). Pairings of TPH2+/- females with TPH2+/+ males showed a slight reduction in pregnancy success to 88% and when TPH2+/- females were mated with TPH2-/- males, success fell to 59%. However, when the female of a mating pair was a TPH2-/- mouse, the success rate dropped more substantially. As shown in Table 1, pairings of TPH2-/- females with TPH2+/- males reduced success to 73%. When both members of a pairing were TPH2-/-, the pregnancy success rate fell to 41%. Despite the observation that TPH2-/- females are preferred by males of both genotypes in inanimate and animate pairings to test sex preference, this does not translate to higher rates of successful matings and offspring birth. Similarly, TPH2-/- males have a significant effect in diminishing mating success when paired with a TPH2+/- or TPH2-/- female.

**Table 1:** Mating success rates in TPH2+/+, TPH2+/- and TPH2-/- mice.

	♀ genotype	♂ genotype	# successful matings	# unsuccessful matings	Total matings	% success
1	TPH2+/+	TPH2+/+	88	11	99	88.9
2	TPH2+/-	TPH2+/-	29	1	30	96.7
3	TPH2+/-	TPH2+/+	7	1	8	87.5
4	TPH2+/-	TPH2-/-	40	28	68	58.8
5	TPH2-/-	TPH2+/-	43	16	59	72.9
6	TPH2-/-	TPH2-/-	79	115	194	40.7

Mating pairs of the indicated genotypes were caged together for 21 days after which the male was removed. From this time forward, the female was housed alone until pups were born or until the passage of an additional 21 days. Matings were scored as successful if pups were born and matings that extended beyond the 21 day post-mating period without births were deemed unsuccessful. Statistical comparisons among rows and the level of significance is indicated below: row 1 different from rows 4 (p < 0.0001), 5 (p = 0.015) and 6 (p < 0.0001); row 2 different from rows 4 (p < 0.0001), 5 (p = 0.008) and 6 (p < 0.0001); row 3 different from row 6 (p = 0.011); row 4 different from rows 1 (p < 0.0001), 2 (p < 0.0001), and 6 (p = 0.011); row 5, different from 1 (p = 0.015), 2 (p = 0.008), and 6 (p < 0.0001); and row 6, different from rows 1 (p < 0.0001), 2 (p < 0.0001), 3 (p = 0.011), 4 (p = 0.011), and 5 (p < 0.0001).

### Pheromone detection and odorant signal transduction determinants of sexual preference in TPH2-/- males

**Fig 4:**
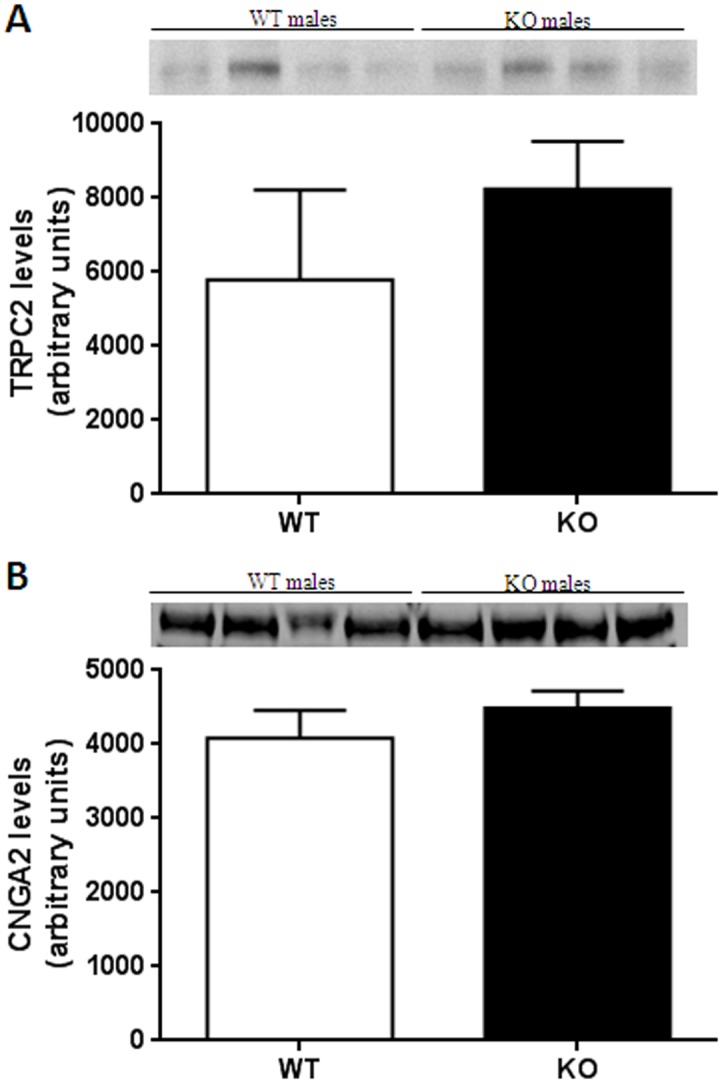
Expression levels of TRPC2 and CNGA2. (A) Immunoblot analysis of TRPC2 in VNO tissue from WT and KO male mice, (B) immunoblot analysis of CNGA2 in MOE tissue from WT and KO male mice. Expression levels of TRPC2 and CNGA2 were normalized to GAPDH levels. Data presented in the bar charts are expressed in arbitrary units and are the mean ± SEM for groups containing 5–6 WT and 4–5 KO males for each tissue. The insets above each bar chart are representative immunoblots for each protein.

A null mutation in the gene for TRPC2 [46,48] or surgical removal of the VNO [47] results in bisexual mounting behavior in male mice. Therefore, we tested male TPH2+/+ and TPH2-/- mice for the expression levels of TRPC2 in VNO tissue. The immunoblotting results presented in Fig. 4A indicate that TPH2-/- mice have the same levels of expression of VNO TRPC2 as TPH2+/+ males. It has also been shown that the MOE-specific protein CNGA2 is an important determinant of sexual discrimination because mice lacking the gene for this protein lose their preference for females over male mice [49]. In view of this observation, we determined CNGA2 expression levels in the MOE of TPH2+/+ and TPH2-/- mice and the results are presented in Fig. 4B. It can be seen that there is no difference in the expression of CNGA2 between male TPH2+/+ and TPH2-/- mice. Taken together, the results in Fig. 4 do not support the possibility that male TPH2-/- mice have deficient expression of VNO and MOE factors that are critical for determining male sex preference. Rather, these data showing normal levels of expression of TRPC2 and CNGA2 is consistent with behavioral observations showing that TPH2-/- male mice retain wild-type levels of sexual preference for females and for female urinary odorants.

## Discussion

The results presented in this paper show clearly that male mice with a genetic depletion of 5-HT retain their preference for females over males and stand in contrast to a recent study reporting that male mice with significant reductions in the number of 5-HT neurons in brain (i.e., *lmx1b* knockout) or with a null mutation in the gene for TPH2 lose their preference for females [1]. With regard to TPH2-/- males specifically, Liu and colleagues showed that these mice displayed shorter latencies to mount and higher frequencies of mounting wild-type males versus females and unlike TPH2+/+ males, TPH2-/- males did not show a preference for the odor of cage bedding or the genital odor of female over male mice [1]. Treatment of TPH2-/- mice with 5-hydroxytryptophan (5-HTP) to replenish brain 5-HT levels restored the aforementioned behavioral deficits to wild-type levels [1]. We also show presently that male TPH2-/- mice do not exhibit hypersexuality by comparison to TPH2+/+ males as reflected in the number of mounts and intromissions. This result stands in contrast to studies showing that pharmacological or dietary depletion of 5-HT facilitates copulatory behavior in rodents [1,12,13,14,15,16,17,18,19].

A number of critical methodological factors could contribute to the discrepancies noted in the roles played by 5-HT in determining sexual behavior and preference. The experimental approach used to gauge sexual preference when offering male subjects the choice between two inanimate sex-related stimuli (i.e., urine scents of a male or an estrous female) or when comparing sexually-directed behaviors in one-on-one pairings of mice (i.e., between male and female or male and male) is straightforward. In these cases, we observed that TPH2-/- males express a preference for females over males to the same extent as TPH2+/+ males. However, when examining sex preference by offering a resident male the simultaneous choice of an intruder male and an intruder female, this grouping of 3 mice is extremely complex and involves far more than the resident male solely expressing a sex choice between the two intruder mice. The intruder male and female mice in this paradigm are also faced with several active choices and do not remain passive in the cage while the resident male makes a choice between them. Certainly, the resident male approaches and eventually mounts the female or the male intruder, or both. We frequently observed that the intruder male will also mount the intruder female while the resident male stands by. If the sexual advances of the resident or intruder male are rejected by the intruder female, it often provokes an attack by the rejected male on the other male. Finally, the resident male, especially TPH2-/- males, attack the intruder male and female without obvious provocation as seen in the resident-intruder test of aggressive behavior [39,41]. For these reasons, we were not surprised that TPH2-/- and TPH2+/+ males showed no sexual preference when 3 mice were in the test cage together, because of competing sexually- and non-sexually directed behaviors being displayed by all 3 mice simultaneously. In an attempt to eliminate the array of behaviors that compete with sexual approach activity in male mice when placed into the presence of two other mice, the intruder mice were placed in the test cage within a small wire cup. This wire cup allows olfactory, auditory and visual contact by the resident male with both intruder mice, and limited tactile contact is possible (e.g., sniffing, touching), but the wire cups prevent mounting, grasping and fighting. This method has been used very successfully in testing social approach in animal models of autistic-like behaviors [40,44,45]. Results of studies using this approach were unequivocal and showed that TPH2-/- males, like TPH2+/+ males, when presented simultaneously with two mice of the opposite sex, expressed a significant preference for investigating female TPH2+/+ and TPH2-/- mice over TPH2+/+ males.

The pharmacological tools used to study the role of 5-HT in determining sexual preference and activity are limited in their specificity and may have contributed to the confusing state of affairs in this area for several reasons. First, early studies showing that reductions in brain 5-HT led to increased sexual activity depended on the use of extremely high doses of the TPH2 inhibitor p-chlorophenylalanine (pCPA). This inhibitor indeed lowers brain 5-HT levels but it also leads to large depletions of dopamine and norepinephrine [50]. pCPA also causes an acute hyperphenylalaninemia in rodents and results in cortical DNA damage as soon as 1 hr after treatment [51]. Drugs that interact with the dopamine and norepinephrine neuronal systems are known to exert powerful effects on sexual behavior in male rodents [52], making it very difficult to ascribe pCPA-induced changes in sexual preference to 5-HT specifically. Second, the use of 5-hydroxytrytophan (5-HTP), the immediate precursor of 5-HT, to reverse pCPA- or genetically engineered-induced deficits in 5-HT levels is problematic. While 5-HTP rapidly increases brain 5-HT concentrations, this effect is not restricted to 5-HT neurons. 5-HTP is converted to 5-HT by the ubiquitous L-aromatic amino acid decarboxylase which is also located in dopamine and norepinephrine neurons. As a result, the effects of 5-HTP on brain chemistry are not selective for the 5-HT neuronal system and lead to large increases in 5-HT in anatomical sites where it is not found normally [53]. When given without a peripheral decarboxylase inhibitor, 5-HTP is also well known to cause a severe, watery diarrhea [54,55,56]. Furthermore, we have shown in preliminary studies with TPH2-/- mice [57] that CNS 5-HT receptors are hypersensitive to a variety of 5-HT agonists, including 5-HTP, which is manifested by the appearance of the 5-HT syndrome. This debilitating neurological syndrome includes head twitches, forepaw treading, tail lashing and hind paw abduction [58]. Both of these effects of 5-HTP would be very disruptive of most ongoing behaviors, to include social and sexual interactions. In addition, any brain developmental deficiencies resulting from the loss of TPH2 [36,59] would not likely be rescued by 5-HTP.

Mice lacking the gene for TPH2 are viable and fertile and, for the most part, have normal morphological and physiological characteristics [23,24,26,27]. Brain development in these mice is essentially the same as seen in TPH2+/+ mice [23,28,35,37,38,39,60] although changes in GABA levels [36] and 5-HT neuronal circuitry formation [59] have been noted. From a behavioral perspective, the TPH2-/- mouse shows intense compulsivity and impulsivity [39], autism-like social communication deficits [40] and exaggerated aggression with decreased levels of anxiety [39,41]. It does not appear that any of these anti-social behaviors exhibited by TPH2-/- males and females (i.e., behavioral disinhibition) alters sexual preference in TPH2-/- males but they may well influence the ability of these mice to achieve successful pregnancies and births. In light of results showing male preference for TPH2-/- females over TPH2+/+ females (Figs. 1B and 3D above) we set up female x male matings that included different combinations of TPH2 genotypes (i.e., TPH2+/+, TPH2-/+ and TPH2-/-). The results showed that this preference did not translate into productive breeding, despite the findings that female TPH2-/- mice were preferred over TPH2+/+ females in choice tests. The least successful pairing was a TPH2-/- female x TPH2-/- male which yielded a mating success rate of 41%. All pairings of TPH2+/+ and TPH2+/- mice were highly successful (89–97%). Pairings in which one partner was TPH2-/- were intermediate in mating success rates (58–72%) by comparison to the highly successful pairings of TPH2+/+ or TPH2+/- mice and the much lower rates seen in pairs of TPH2-/- mice. It was not possible in these studies to determine if a difference existed between TPH2-/- males and TPH2-/- females in contributing to the lowered pregnancy rates. It may well be the case that the behavioral disinhibition phenotype of TPH2-/- mice does not influence sexual activity and preference, but it does interfere with the successful consummation of mating in these mice. Our data have also established that when TPH2-/- female mice do achieve pregnancy, litter sizes are the same as litters of TPH2+/+ females (data not shown), suggesting that reproductive function is normal in these mice. On the other hand, it has been proposed that 5-HT plays a functional role in sperm physiology [61]. Female reproductive tissues also contain 5-HT which is thought to play numerous roles in embryonic development [62,63] and TPH2 expression has even been demonstrated in early pre-implantation embryos [64]. Therefore, it may be the case that TPH2-/- mice do have subtle alterations in reproductive function but much more additional work is needed to specify these potential changes precisely.

In summary, we conclude that a genetic depletion of 5-HT from brain does not have an effect on sexual preference or activity. Previous studies that have implicated 5-HT signaling in these behaviors used pharmacological approaches (i.e., pCPA and 5-HTP) that lack specificity for the 5-HT neuronal system and which have numerous, serious adverse effects, both of which make it difficult to attribute observed changes in behavior to 5-HT. The use of inanimate sexual stimuli (urine scents) and pairings between single male and female mice showed clearly that TPH2-/- males prefer females over males. This preference appeared to be lost when TPH2-/- and TPH2+/+ males were given the choice of interacting with a male or female intruder mouse simultaneously. When competing, non-sexually related behaviors that were displayed among 3 interacting mice were controlled by placing the two intruder mice in wire cups, the preference of male TPH2-/- mice for females was affirmed. TPH2-/- females were preferred over TPH2+/+ females in most tests but this effect did not transfer into fruitful matings in TPH2-/- mice. Finally, the finding of normal expression of TRPC2 in the VNO and of CNGA2 in the MOE of TPH2-/- males alone supports the conclusion that the sexual preference of TPH2-/- males for females over males is intact.
